# Improving memory for unusual events with wakeful reactivation

**DOI:** 10.3389/fpsyg.2023.1092408

**Published:** 2023-03-28

**Authors:** Arit Glicksohn, Ladan Shams, Aaron R. Seitz

**Affiliations:** ^1^Department of Psychology, University of California, Los Angeles, Los Angeles, CA, United States; ^2^Department of Psychology, University of California, Riverside, Riverside, CA, United States; ^3^Department of Psychology, Northeastern University, Boston, MA, United States

**Keywords:** episodic memory, multisensory memory, memory reactivation, memory tagging, consolidation

## Abstract

Memory consists of multiple processes, from encoding information, consolidating it into short- and long- term memory, and later retrieving relevant information. Targeted memory reactivation is an experimental method during which sensory components of a multisensory representation (such as sounds or odors) are ‘reactivated’, facilitating the later retrieval of unisensory attributes. We examined whether novel and unpredicted events benefit from reactivation to a greater degree than normal stimuli. We presented participants with everyday objects, and ‘tagged’ these objects with sounds (e.g., animals and their matching sounds) at different screen locations. ‘Oddballs’ were created by presenting unusual objects and sounds (e.g., a unicorn with a heartbeat sound). During a short reactivation phase, participants listened to a replay of normal and oddball sounds. Participants were then tested on their memory for visual and spatial information in the absence of sounds. Participants were better at remembering the oddball objects compared to normal ones. Importantly, participants were also better at recalling the locations of oddball objects whose sounds were reactivated, compared to objects whose sounds that were not presented again. These results suggest that episodic memory benefits from associating objects with unusual cues, and that reactivating those cues strengthen the entire multisensory representation, resulting in enhanced memory for unisensory attributes.

## Introduction

Which memories do we remember and which do we forget? Can we influence this process by rendering certain events and objects more memorable? Memory consists of multiple processes, from encoding information, consolidating it into short-and long-term memory, and later retrieving relevant information. Classical research revealed that maintaining the same context when first encountering information and later retrieving it enhances memory (Tulving and Thomspon, 1973). One form of context is multisensory information, where multisensory encoding improves later retrieval of the *unisensory details* of these memories, such as better remembering images previously presented with sounds (Thelen and Murray, 2013; Thelen et al., 2015; Duarte et al., 2022; but see Pecher and Zeelenberg, 2022). The benefits of multisensory memory can arise from several mechanisms: One proposed mechanism is “*redintegration*,” whereby semantic multisensory representations are created during encoding, and later activated by their unisensory stimuli (Von Kriegstein and Giraud, 2006; Shams and Seitz, 2008; Shams et al., 2011). Another possibility is that multisensory representations modify the unisensory representations themselves, rendering them more precise and accessible for retrieval (Shams and Seitz, 2008; Shams et al., 2011).

Following the encoding of new knowledge or skill, a consolidation process occurs whereby information is transferred over time into long-term memory. The consolidation process is considered an off-line memory process during which memories are strengthened, occurring at wake and sleep after learning. An emerging approach termed *targeted memory reactivation* (TMR) reveals that when sensory components of the multisensory representation (such as sounds or odors) are “reactivated” during sleep, a replay of associated memories occurs, facilitating the later retrieval of the entire memory representation (Rudoy et al., 2009; Oudiette and Paller, 2013). In one of the original studies (Oudiette and Paller, 2013), the procedure consisted of a learning period, whereby participants were presented with visual images and their matching sounds (e.g., cat-“meow”) appearing at different locations on a computer screen. Participants were instructed to learn the locations of the objects. The visual images had different values associated with them, with half of the images having a high value and half a low value. Following learning, half of the participants went to sleep in the lab, and were unknowingly exposed to the sounds from the learning phase. During a test phase the next day, all participants were better at recalling the location of the high-value images compared to low-value images. Importantly, for participants who experienced the reactivation of sounds, the low-value images associated with reactivated cues were better remembered compared to low-value images not associated with such cues. This finding reveals that “weaker” memories – events with initial lower memory strength – particularly benefit from reactivating their unisensory components (Oudiette et al., 2013; Oudiette and Paller, 2013). Studies across a range of domains have shown that TMR can be beneficial not only for episodic memory, but for language and skill learning as well (Rudoy et al., 2009; Antony et al., 2013; Hu et al., 2020; Laurino et al., 2022).

Are some memories reactivated while others not? Do some memories benefit from reactivation to a greater degree than others? *Memory tagging* refers to the process whereby new information is tagged for its potential importance or value. Memories tagged as valuable are thought to be rehearsed over time, with underlying neural circuits being strengthened, and certain events rendered more memorable (Oudiette et al., 2013). Tagging is influenced by multiple factors, such as attention, intention, emotion and reward. One potent form is *novelty-based tagging*, whereby novel attributes associated with an object (e.g., a visual image paired with a novel sound rather than a repeating sound), enhances object memory. The “oddball” is thought to trigger an attentional mechanism, resulting in enhanced object processing (Kim and McAuley, 2013; Cohen Hoffing and Seitz, 2015; Liao et al., 2016). Cowan et al. (2021) further propose that memory tagging enhances consolidation, whereby *goal-relevant* information, considered particularly rewarding or valuable for achieving one’s goals, is “tagged” for future consolidation. Another type of memory tagged for consolidation are *“weaker”* memories – information that is initially weakly encoded or learned (Tambini et al., 2017; Schapiro et al., 2018; Denis et al., 2021).

Memories tagged for consolidation may be the ones that benefit the most from TMR. Events and objects that are novel and unpredicted may be a good candidate for TMR, as they signal a rapid change in the environment, making them both salient and valuable in deciphering a new situation. However, novel stimuli may be also “weaker,” as they are not easily encoded into pre-existing schemas, and may need further processing. To test this hypothesis, we compare the influence of reactivation on novel versus normal (predicted) stimuli, and test memory for different attributes of the multisensory representation.

### Sensory cues

What types of sensory cues are most beneficial for TMR? Sensory cues strongly associated with memories are particularly beneficial, such as semantically-associated visuals and sounds (e.g., cat-“meow”; Oudiette et al., 2013). Sound melodies matched to simple action patterns are also helpful (Antony et al., 2013). New associations between senses can also be formed during a short learning period (Rasch et al., 2007; Vargas et al., 2019), especially with potent stimuli such as odor. In their seminal study, Rasch et al. (2007) presented participants a single scent of a rose when learning the locations of objects. During sleep, half of the participants were presented with the rose scent. During a memory test, participants were presented with objects without the odor, and asked to recall the locations of the objects. These participants showed improved spatial memory compared to participants who did not experience the reactivation of the single odor. These findings suggest that the odor served as a context-cue for all objects. Yet, in most studies, the test phase includes the presentation of the reactivated sensory cue, serving as a potent retrieval cue. This leaves open the question of whether reactivation alone can modify the multisensory representation, modifying its components such that it is easier to freely recall them, even in the absence of reactivated cues. We address this question in our study.

### Wakeful TMR

Memory improvement also occurs when sensory cues are reactivated during a resting period, and not just sleep (Oudiette et al., 2013; Tambini et al., 2017). For example, after participants learnt the locations of objects, they performed a simple repetitive task whereby some visual objects appeared. This visual reactivation during another task resulted in improved memory for the locations of the objects (Tambini et al., 2017). The authors suggest that reactivation benefits memory consolidation during times in which the hippocampus is not engaged in coding novel information, such as a restful period or sleep. However, the conditions that enable successful TMR during wakefulness are not yet clear, as other studies have not found such a benefit (Rudoy et al., 2009; Diekelmann et al., 2011; Schreiner and Rasch, 2015). For example, vocabulary learning was enhanced when words were reactivated during sleep, but not during active and passive waking (Schreiner and Rasch, 2015). Diekelmann et al. (2011) suggest that TMR is not a unitary phenomena, but rather underlied by different mechanisms and brain areas operating during sleep and wakefulness. One proposal is that reactivation during sleep stabilizes and strengthens memories, while reactivation during wakefulness does the opposite by destabilizing memories, allowing newer and more relevant information to override these memories. We theorize that unusual events alert us to changing environmental circumstances and expectations, and that wakeful reactivation can be particularly beneficial in modifying these memories.

**Figure 1:**
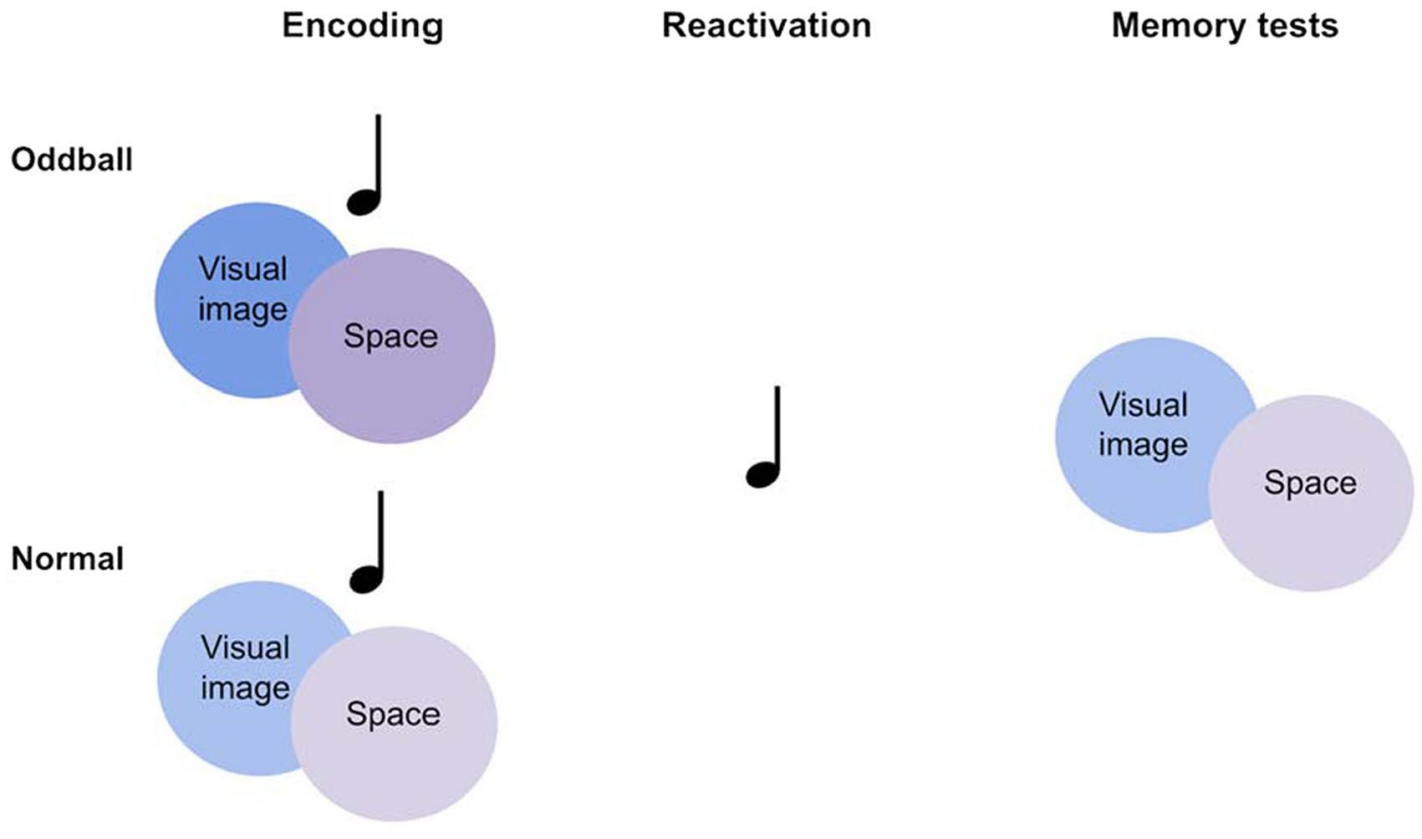
Graphic representation of the multisensory oddball memory task.

To address this theory, we tested the following hypotheses: Wakeful reactivation of novel and unusual events will enhance encoding of these events compared to normal events, in line with previous research (Hunt and McDaniel, 1993). Importantly, memory for unusual events will be improved by coupling these events with sensory cues during encoding, and later reactivating these cues. By forming a rich multisensory representation, reactivating a component of this representation will benefit memory for other components as the entire multisensory representation is strengthened. While prior studies tested memory in the presence of the sensory cues that were reactivated during sleep or wakefulness, we test visual memory without sound cues, hypothesizing that the shared multisensory representation is evoked. We conducted two experiments in which participants were first presented with audiovisual objects at different locations, followed by a replay of sounds. Participants were then tested on their memory for visual and spatial information in the absence of sounds. This method addresses the question of whether reactivation in one sensory modality leads to memory benefits in another modality. In Experiment 1, audiovisual objects consisted of everyday objects coupled with their corresponding sounds, with “oddball” objects consisting of unusual objects and sounds. In Experiment 2, everyday objects were coupled with repeating sounds, with “oddballs” created by associating certain objects with an irregular sound. During both experiments, participants completed a cognitive ability task in computerized form (Raven’s Advanced Progressive Matrices) to further test the possible relations between memory improvement and cognitive ability (see Figure 1).

## Experiment 1: Audiovisual tagging

### Method

#### Participants

A total of 78 participants were run. Nine participants were removed due to exhibiting no correct responses in some conditions during the memory test phase (thus creating a missing design), resulting in 69 participants. All participants had normal or corrected-to-normal visual acuity and received course credit for a 1-h session. All participants gave written informed consent, approved by the University of California, Riverside Human Research Review Board.

#### Apparatus and stimuli

An Apple Mac Mini running Matlab (MathWorks, Natick, MA, United States) and Psychophysics Toolbox Version 3.014 (Brainard, 1997; Pelli and Vision, 1997) was used for stimuli generation and experiment control. Stimuli were presented on a ViewSonic PF817 monitor with a 1,600 × 1,200 resolution, and a refresh rate of 100 Hz.

#### Design

**Figure 2:**
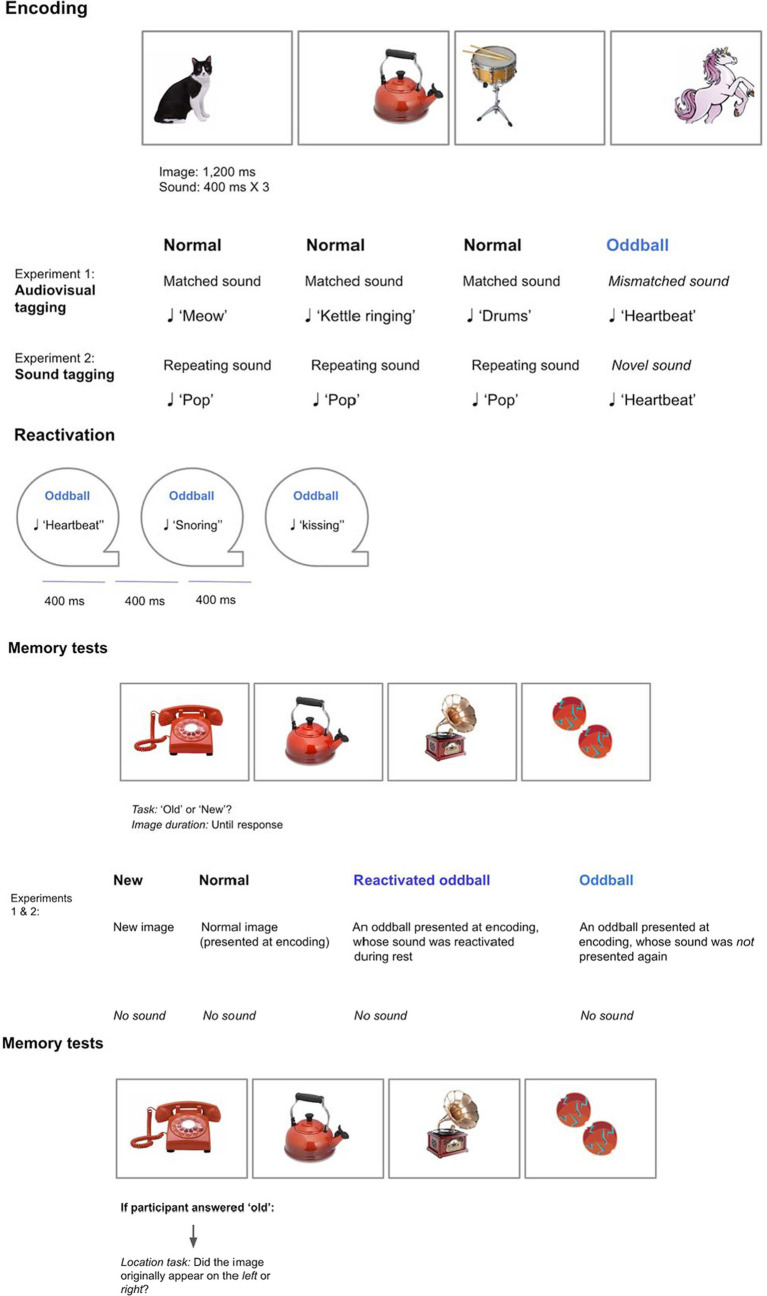
Graphic depiction of Experiments 1–2.

A 2×2 within-participants design was employed with the following factors: *Stimuli* (oddball/normal) and *Reactivation* (yes/no). We created an *oddball memory task*, consisting of three parts: Encoding, reactivation, and memory tests (see Figure 2). All of the tasks were performed successively within a single session.

#### Encoding phase

We created *audiovisual tagging*:

- *Normal* visual stimuli appeared with their matching sounds, such as animals with their vocals, and musical instruments with their notes. Stimuli belonged to seven categories, each category consisting of 12 images, for a total of 98 trials (see Supplementary Appendix I for full stimuli description).
- *Oddballs* were created by presenting a mismatching object for each category (e.g., animals: *unicorn*, musical instruments: *gramophone*). These objects were also paired with mismatched sounds (e.g., unicorn—heartbeat sound, gramophone—kissing sound). Each category included two oddballs, resulting in a total of 14 oddball trials.

Overall there were 112 trials. Participants were presented with a stream of visual and auditory stimuli. Each image (normal or oddball) appeared randomly either to the left or right of a fixation point for 1,200 ms. Each normal sound appeared at the onset of the visual image for 400 ms. In contrast, an oddball sound was repeated three times to reinforce the sound, with no pause between sounds.

We created two oddball conditions:

- *Category oddballs (N = 56).* Stimuli presentation was as follows: There were two presentation blocks, with the seven categories appearing in successive order (e.g., category 1–category 2, etc.). The order of the categories was chosen randomly for each presentation. Overall, each category consisted of 16 images, 14 normal images, and 2 oddballs. The images for each category presentation were also chosen randomly, with each image appearing once during encoding. However, the number of images for each category presentation was varied. There are seven possible options to split images (e.g., 7–9, 10–6, 11–5, etc.), and each category was randomly associated with an option. This meant that participants could not predict the length of each category. The oddball image appeared randomly during the category presentation, with the constraint that it did not appear in the first two places. The intention was that the oddball will “pop-out” from the category (following the method of Cohen Hoffing and Seitz, 2015). Each category appeared in one location of the screen (either left or right to the fixation point) chosen randomly.
- *Random oddballs (N = 22).* Another approach was to present oddballs randomly, so that the distinct perceptual features of the stimuli will pop-out regardless of category. Oddballs appeared amidst random objects—images were randomly chosen from the seven categories – and changed location on each trial, with the constraint that an oddball was preceded by at least three normal stimuli. All other presentation attributes were similar to those of the *category oddballs* condition.

In both conditions, to ensure participants engaged with the stimuli, they were asked to perform two successive tasks: (i) Judge whether the category of the present object matched that of the previous one (*n*−1 task), pressing 3 for the same category, and 4 for a different category. (ii) To verify our oddball stimuli, participants were then asked to indicate for each sound whether it was surprising or not, pressing the spacebar if the sound was surprising.

The conditions were run sequentially across two academic quarters. We recruited available participants for each condition, with a non-optimal end result of an unequal number of participants between conditions. Importantly, though, this is a within-participant study, enabling a direct comparison of the main *reactivation* manipulation, as will be described shortly.

#### Cognitive ability task

Participants completed an abridged version of Raven’s advanced progressive matrices in computerized form (12 questions; based on Kubricht et al., 2017), with questions presented in order of increased complexity. There was a time limit of 12 min, after which the study proceeded to the next part automatically. Participants were presented with 2 practice trials prior to completing the test questions. The score is calculated as percent correct.

#### Reactivation phase

Participants were presented with a stream of 84 sounds *via* headphones. Each sound appeared for 400 ms, with a short pause between sounds that lasted 400 ms. The sounds appeared in random order for each participant. The following sounds were reactivated:

- *Re-activated oddballs:* one oddball sound per category was randomly chosen for each participant, with each sound repeating three times (21 presentations in total).
- *Re-activated normal stimuli:* three normal sounds per category were randomly chosen for each participant, with each sound repeating three times (63 presentations in total).

During the sound presentation, participants were asked to complete simple word puzzles, with the following instructions: “You will hear a series of sounds. You will also complete a word search on paper. Please keep your headphones on during the entire part.” The word puzzles consisted of a list of words, for example, geography words, and participants were asked to find the words in a big table of letters (see Supplementary Appendix I for an example). There was no semantic overlap between the puzzle words and the sounds presented during the memory phases. This phase lasted approximately 5 min. Recognition accuracy was measured.

#### Recognition test

Images appeared at the middle of the screen without sound. Participants judged each image as “old” or “new.” There were five stimuli conditions, totaling 112 trials:

- *Re-activated oddballs* (seven trials): Oddballs presented during encoding, and whose sounds were presented later again.
- *Non-reactivated oddballs* (seven trials): Oddballs presented during encoding only.
- *Re-activated normal images* (21 trials): Normal stimuli presented during encoding, and whose sounds were presented later again.
- *Non-reactivated normal images* (21 trials): Normal stimuli presented during encoding only.
- New images that did not appear during the encoding phase (56 trials).

Each image appeared until the participant responded. To capture different forms of recognition memory, participants answered using a *confidence scale*. The instructions were as follows: “You will see a series of pictures. Half of the pictures appeared in Part I (Old pictures), and half will appear for the first time (New pictures). For each picture, please answer whether the picture new or old? Press 1-Old Remember, 2-Old Familiar, 3-New.”

#### Location test

If participants indicated that an image was “old,” they were asked to recall the original location of the image during encoding using a second confidence scale. We hypothesized that object memory and object-location memory may be differently sensitive to the experimental manipulation, and therefore assessed confidence separately for location recall as well. The instructions were as follows: “If the picture is Old, did it appear in the Left or Right side? Press 1—Left remember, 2—Left familiar, 3—Right familiar, and 4—Right remember.” Location accuracy was measured.

Statistical analyses were conducted with the programming language R, with the tidyverse package (v1.3.2; Wickham et al., 2019), ggplot2 (v3.3.6; Wickham, 2016), colorspace (v2.0–3; Zeileis et al., 2020), apex (v1.1–1; Singmann, 2022), emmeans (v1.7.5; Lenth, 2017), and psycho (v0.6.1, Makowski, 2018). We examined accuracy (e.g., percent correct). Outliers were determined as values below Q1 − 1.5 IQR, and above Q3 + 1.5 IQR across conditions, and were excluded from further analysis. For full descriptive statistics of the encoding phase, see Supplementary Appendix II.

### Results

#### Recognition test

**Figure 3:**
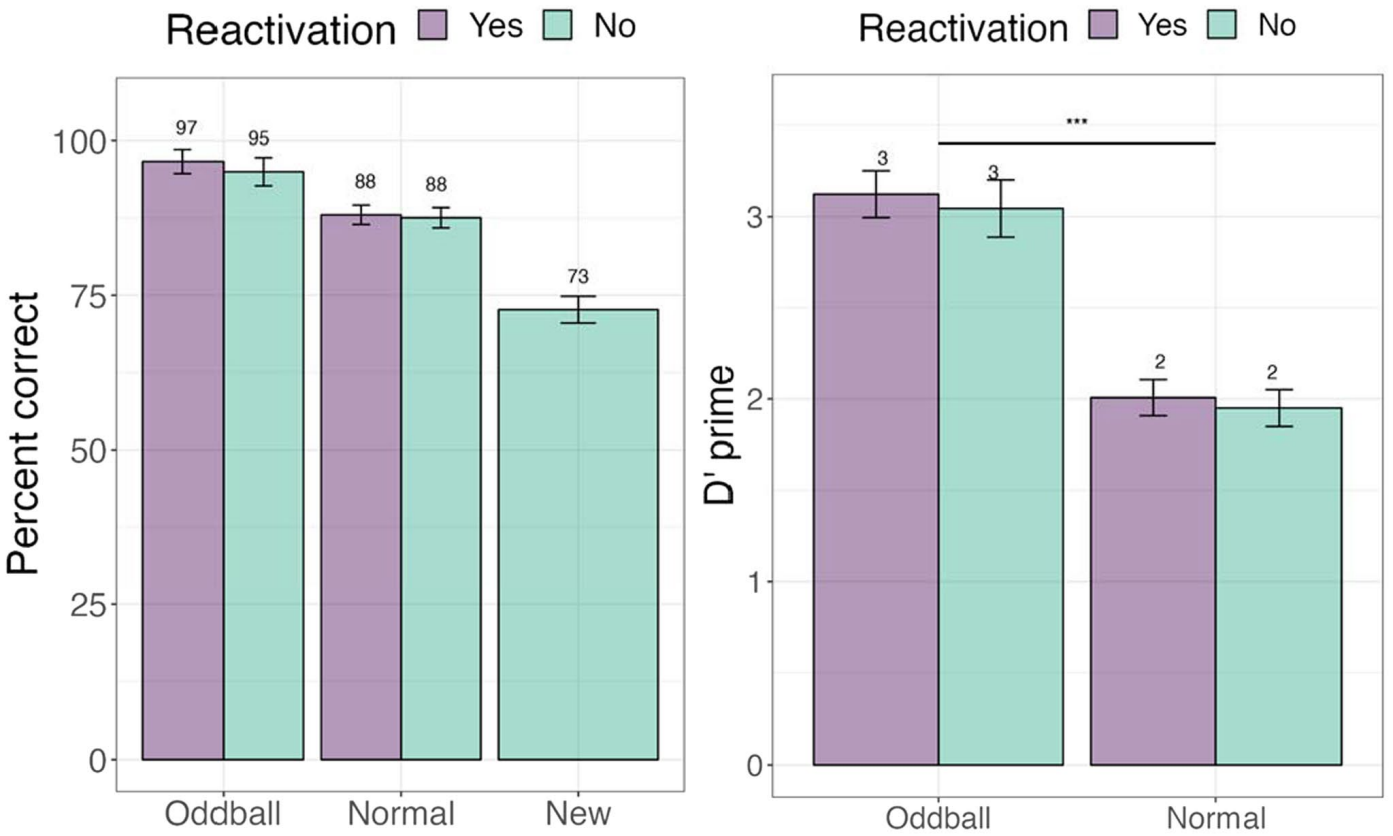
Percent correct and d’-prime values for the visual recognition test in Experiment 1. ***The significance of 0 < 0.001. Error bars represent standard errors of the mean.

We first examined whether tagging led to an improvement in memorization of the images. To address this, we examined accuracy (e.g., percent correct) in the recognition test (see Figure 3). We further calculated signal detection measures—d-prime (sensitivity) and c (criterion) to account for possible response biases. A within-participant Analysis of Variance (ANOVA) was conducted for d-prime, followed by planned contrasts with *stimuli* (oddball/normal) and *reactivation* (yes/no) factors. There was a significant main effect of *stimuli*, *F*(1, 68) = 48.987, *p* < 0.0001, following a Greenhouse–Geisser correction for departure from sphericity. Pairwise contrasts with holm adjustment for multiple comparisons reveal that participants were more accurate at recognizing oddballs than normal stimuli, mean difference = 10.832, *t*(68) = 10.832, *p* < 0.0001. The *reactivation* factor was non-significant, *F*(1, 68) = 0.65, *p* = 0.4. These results establish our memory task as successful in creating highly memorable audiovisual oddballs. For full descriptive statistics see Supplementary Appendix III.

##### Recognition confidence

**Figure 4:**
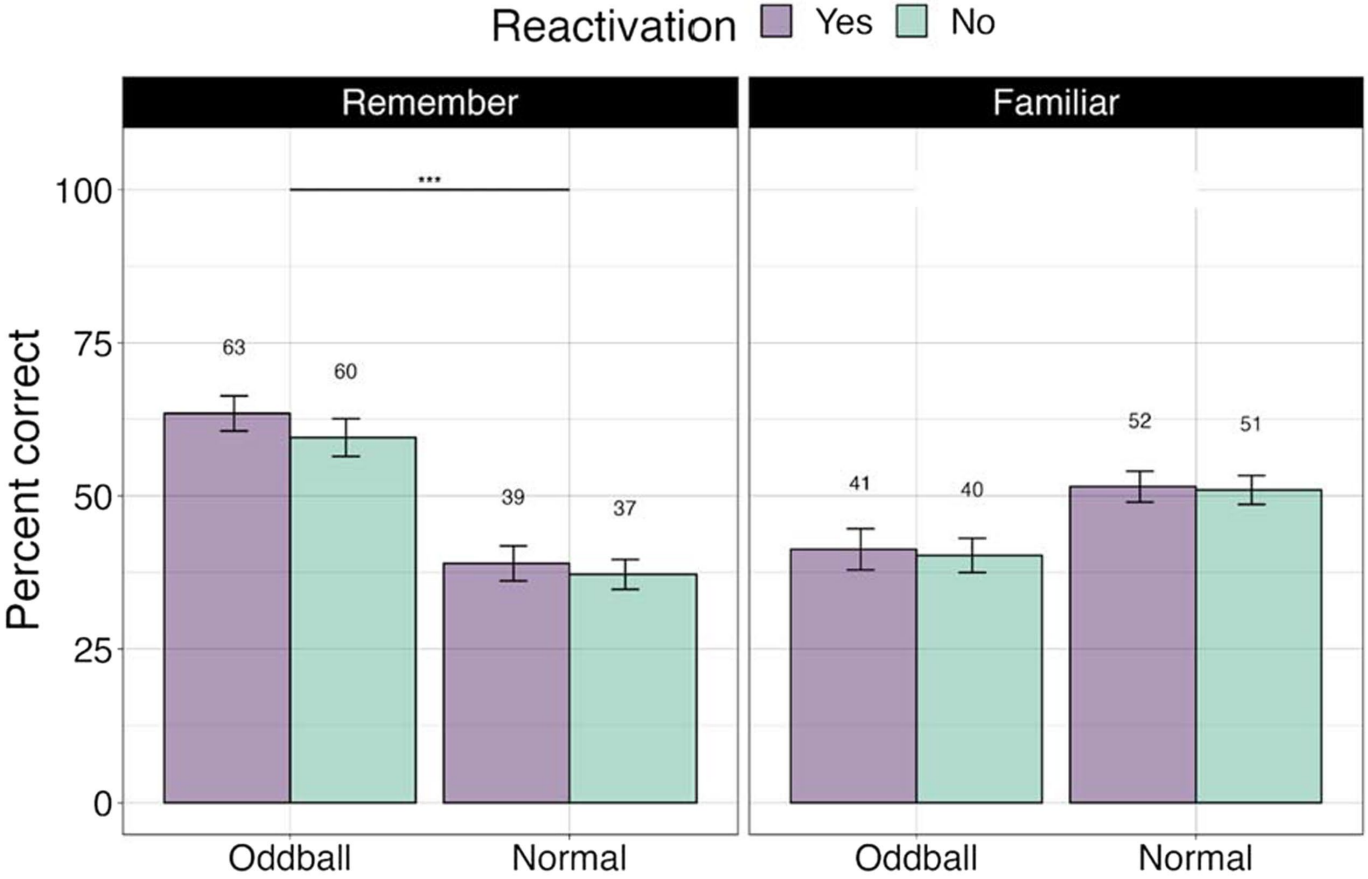
Percent correct for the visual recognition test by self-reported confidence levels in Experiment 1. ***The significance of 0 < 0.001. Error bars represent standard errors of the mean.

To ascertain whether there were differences between stimuli with different levels of memory quality (stimuli that participants explicitly remembered versus were only familiar with), we conducted a 3×2 ANOVA, with *stimuli* (oddball/normal), *reactivation* (yes/no), and *confidence* (remember/familiar) factors. We note that 19 participants were removed from this analysis due to missing values (not all conditions had “remember” or “familiar” responses). There was a significant *stimuli X confidence* effect, *F*(1, 49) = 72.36, *p* < 0.0001, with participants showing greater accuracy for remembered oddballs compared to remembered normal stimuli, or any familiar stimuli. Pairwise contrasts with holm correction reveal that participants were more accurate at recalling the location of ‘remember’ oddballs compared to “familiar” oddballs, mean difference = 19.3, *t*(49) = 4, *p* = 0.0006. Similarly, they were better at recalling “remember” oddballs compared to “remember” normal images, mean difference = 10.27, *t*(49) = 10.27, *p* = 0.0001 (see Figure 4). This result suggests that accuracy and self-report confidence are well matched in this task, with accuracy largely based on recollection-based recognition memory rather than familiarity (Yonelinas et al., 2022). The *reactivation* factor was non-significant, *F*(1, 49) = 0.44, *p* = 0.5. *Stimuli × reactivation* interaction was non-significant as well, *F*(1, 49) = 0.59, *p* = 0.45.

#### Location test

**Figure 5:**
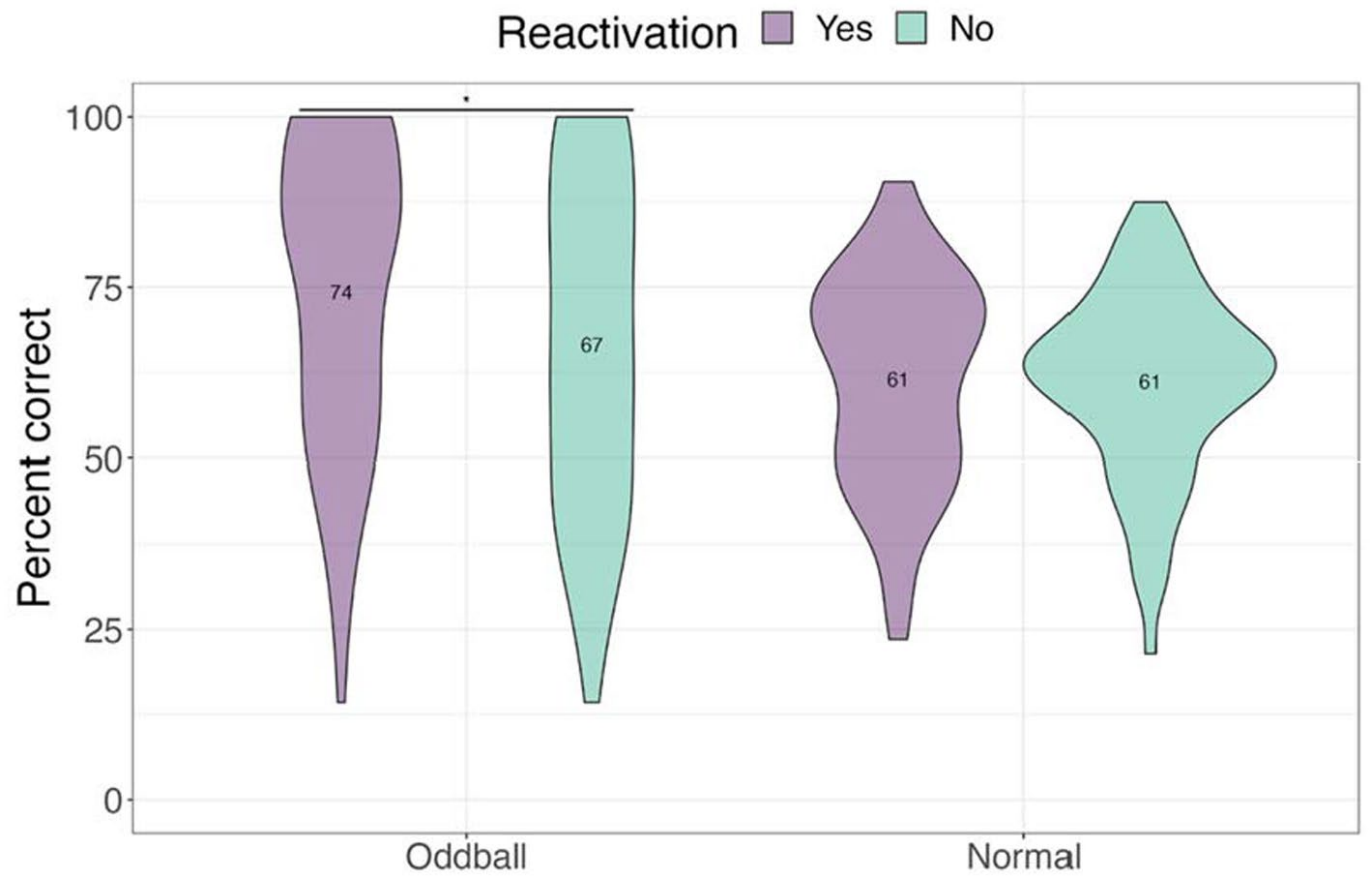
Mean accuracy for the location test in Experiment 1. The violin plot is a mirrored density plot with the kernel density estimates on each side (a violin plot combines boxplot and density plots into a single plot). *The significance of *p* < 0.05.

We next examined performance on the location task to test whether tagging improved contextual knowledge of the stimuli (see Figure 5). Only trials with correct answers on the recognition test were considered. A 2×2 ANOVA with *stimuli* and *reactivation* factors revealed a significant *stimuli by reactivation* interaction, *F*(1, 68) = 4.13, *p* = 0.046, following a Greenhouse–Geisser correction. Participants were better at recalling the location of *reactivated oddballs* compared to *non-reactivated oddballs* in the subsequent absence of sounds, mean difference = 7.74, *t*(68) = 2.5, *p* = 0.015. This finding is consistent with our hypothesis that reactivation will benefit spatial memory for oddballs.

##### Oddball conditions

**Figure 6:**
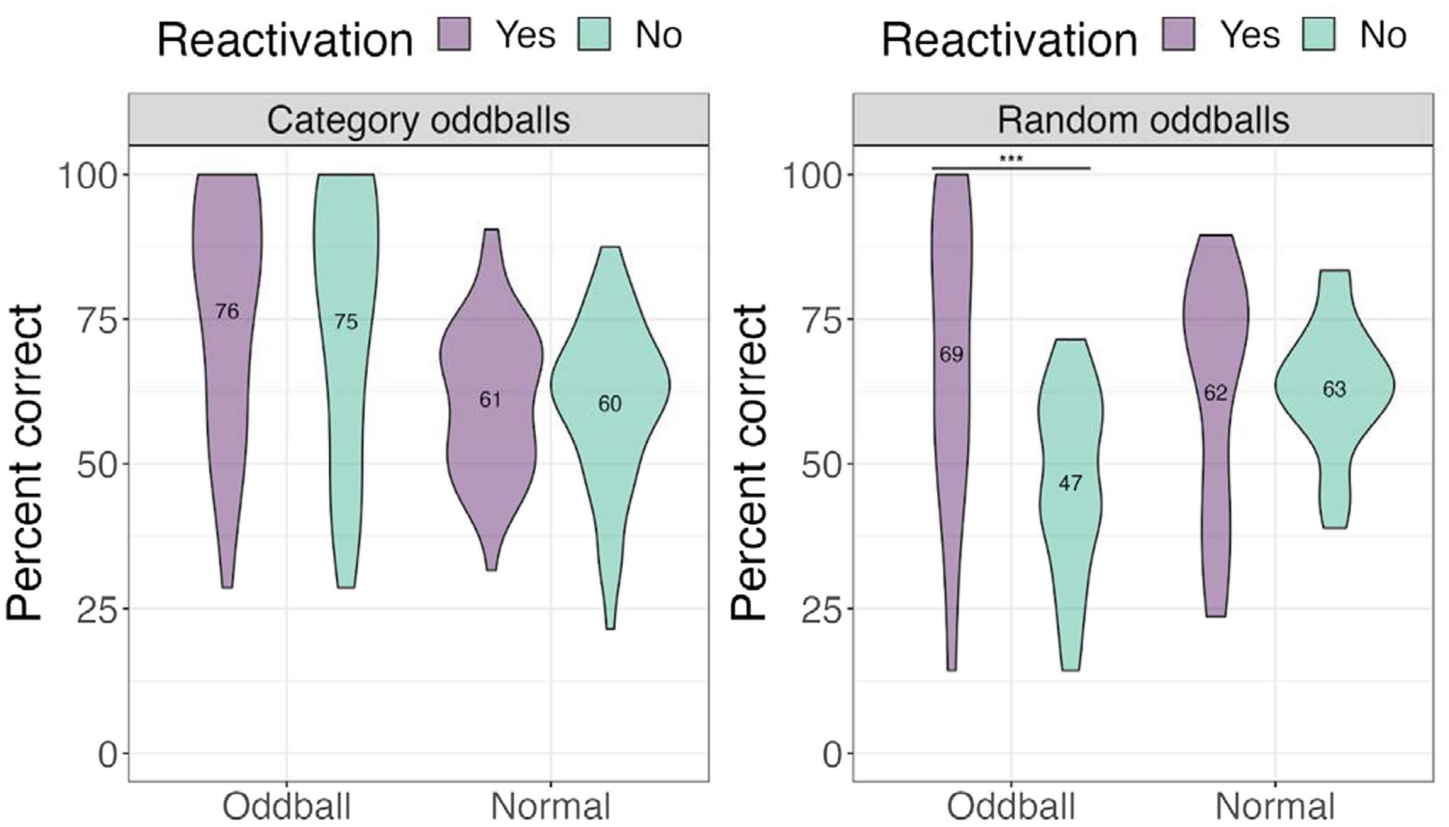
Percent correct for the location test for different oddball conditions in Experiment 1. ***The significance of *p* < 0.001.

We performed a 3×2 mixed ANOVA with a between-participants factor*—oddball condition* (category oddballs/random oddballs), and two within-participant factors—*stimuli* (oddball/normal) and *reactivation* (yes/no), with location accuracy as the dependent variable. There was a significant *condition × stimuli × reactivation* effect, *F*(1, 67) = 8.15, *p* = 0.019. Computing a two-way interaction for each condition level revealed a statistically significant interaction of stimuli and reactivation for *random oddballs*, *F*(1, 67) = 25.479, *p* < 0.001, with participants worse at recalling the locations of *random oddballs* that were not reactivated compared to those that were reactivated (see Figure 6). This result suggests that oddballs attract the most attention when they cannot easily be tied to a familiar stimuli category, and that reactivating the oddball events *via* sound improved spatial memory.

##### Location confidence

**Figure 7:**
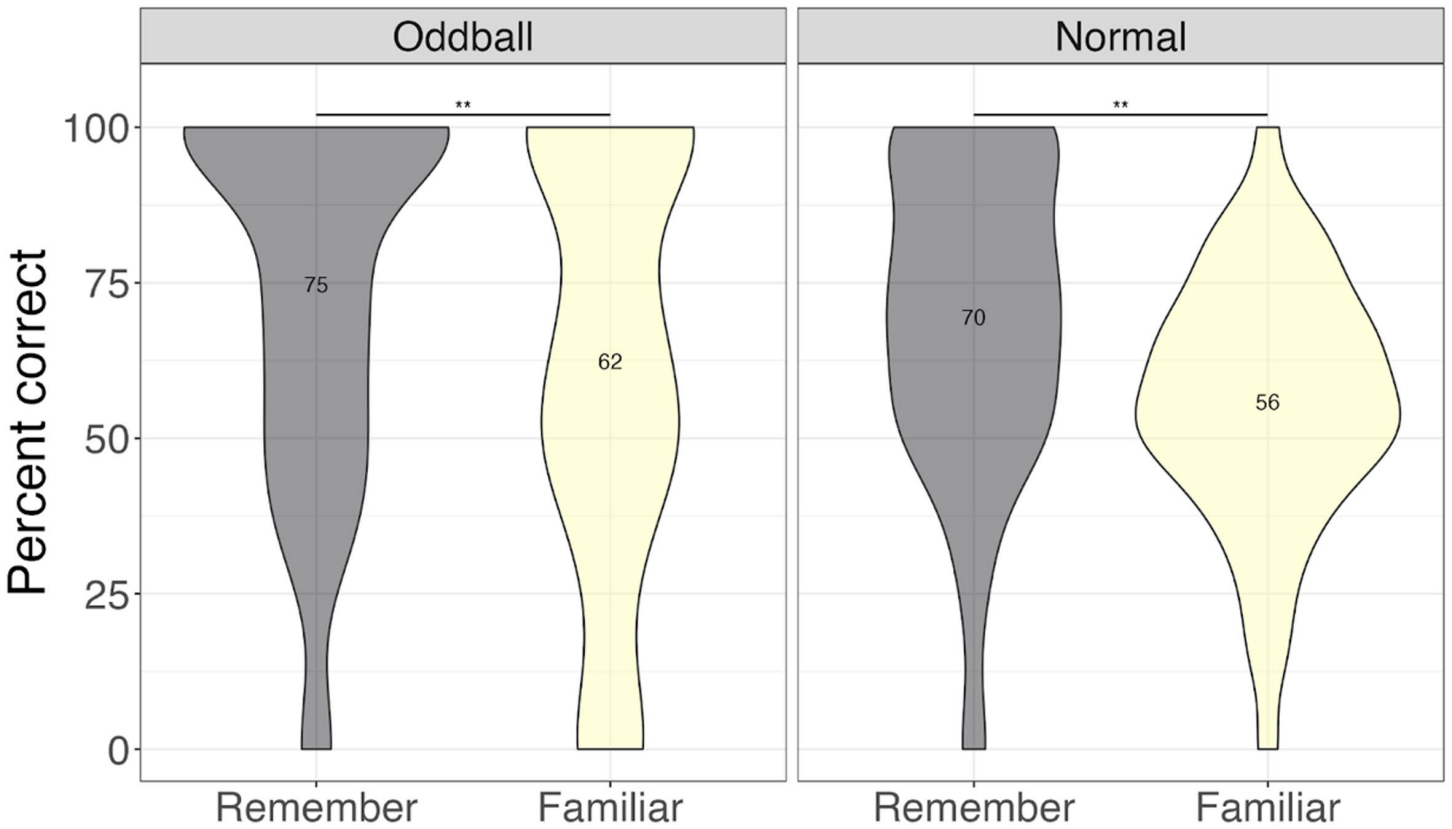
Percent correct for location test by self-reported confidence levels in Experiment 1.

To ascertain whether there were differences between stimuli with different levels of memory quality, we conducted a 3×2 ANOVA, with *stimuli* (oddball/normal), *reactivation* (yes/no), and *confidence* (remember/familiar) factors. We note that 20 participants were removed from this analysis due to missing values (not all conditions had “remember” or “familiar” responses). There was a significant *stimuli* effect, *F*(1, 49) = 5.06, *p* = 0.028, with participants being more accurate at recalling oddball locations than normal stimuli locations. Importantly, there was a significant *confidence* effect, *F*(1, 49) = 29.73, *p* < 0.0001. Pairwise contrasts with holm correction reveal that participants were more accurate at recalling the location of “remember” oddballs compared to “familiar” oddballs, mean difference = 12.5, *t*(49) = 3.46, *p* = 0.0045. Similarly, they were better at recalling “remember” normal images compared to “familiar” normal images, mean difference = 14.35, *t*(49) = 4.8, *p* = 0.0001 (see Figure 7).

##### Cognitive ability

There were no meaningful correlations between general cognitive ability measured as accuracy on Raven’s advanced progressive matrices, sensitivity (d’prime) in the recognition test, and accuracy in the location test. This suggests that the oddball memory task is tapping semantics and long-term memory, while general cognitive ability is associated with working memory ability (Süß et al., 2002; Pahor et al., 2022). See Supplementary Appendix IV for full descriptive statistics (Figure 7).

### Summary

When presented with normal or oddball audiovisual events, participants were better at remembering oddballs on a recognition test. Performance was particularly high, perhaps creating a ceiling effect for any other condition differences to occur. High recognition is mostly due to explicit recognition of the event, and not just mere familiarity, as evident in self-report confidence ratings.

The critical finding of this study is that participants better remembered the location of objects whose associated sounds were reactivated during a wakeful consolidation period, compared to objects whose sounds were not reactivated. Even though object location was encoded incidentally during the first phase, sound reactivation boosted memory for this attribute. Our results further suggest that an encoding stage with random presentation of images compared to one where images appeared in chunked categories yielded a memory benefit for the oddballs, presumably because they were particularly noticeable in the former presentation. Together, these results suggest that memory is improved for rare audiovisual events, and that reactivating a sensory component of the event is enough to enhance memory for other associated attributes.

A key attribute of our memory task is a reliance on existing semantic associations in the case of normal events, with oddballs created by associating a mismatching sound to a rare category member. To better understand what characteristics of the stimuli are necessary to find this effect, we Experiment 2 examines whether sound oddballs in a perceptual stream are enough to form a multisensory representation that can be later evoked during reactivation and retrieval.

## Experiment 2: Sound tagging

### Participants

A total of 46 participants were run. All participants had normal or corrected-to-normal visual acuity and received course credit for 1-h session. All participants gave written informed consent, as approved by the University of California, Riverside Human Research Review Board.

### Apparatus and stimuli

Similar to experiment 1.

### Design

A within-participants design was employed with *Stimuli* (reactivated oddball /oddball/normal) as factor. We created a second version of the oddball memory task, consisting of three parts: Encoding, reactivation, and memory tests.

### Encoding phase

We created *sound tagging*, whereby visual stimuli appeared with the same repetitive sound (“pop”) for a total of 84 trials. Oddballs were created by pairing a subset of normal visual images with a unique mismatched sound (e.g., “heartbeat”) to create a “pop-out” effect. Each oddball image was paired with a unique mismatched sound, with a total of 28 trials. Each image appeared either to the left or right of a fixation point for 1,200 ms. If the sound was a normal one it appeared once for 400 ms. If the sound was an oddball, it repeated three times in order to reinforce this event. Categories appeared successively, so that the 16 images of the same category appeared successively, before moving on to the next category. Participants performed the same tasks as in Experiment 1.

### Reactivation phase

Participants were presented with a stream of sounds *via* headphones. Each sound appeared for 400 ms, with a short pause of 400 ms. Two sounds per category were randomly chosen for each participant, resulting in 14 oddballs. Since tagging consisted of sounds alone (as opposed to sounds and images), we sought to strengthen reactivation effects by repeating each sound 6 times, for the total of 84 trials. Sounds appeared in random order for each participant.

### Recognition test

Images appeared at the middle of the screen without sound. Participants judged stimuli as “old” or “new” with the same *confidence scale* as in Experiment 1.

There were four stimuli conditions, totaling 112 trials. The following stimuli appeared during the test:

- *Reactivated oddballs:* Images whose sounds appeared during reactivation (14 trials)
- *Non-reactivated oddballs:* Images that appeared during encoding, but their sounds did not appear during reactivation (14 trials).
- *Non-reactivated normal images*: Images that appeared during the encoding phase (28 trials).
- *New:* Images that did not appear during the encoding phase (56 trials).

### Location test

If participants indicated that an image was “old,” they were asked to recall the original location of the image using the same *confidence scale* as in Experiment 1.

### Results

#### Recognition test

**Figure 8:**
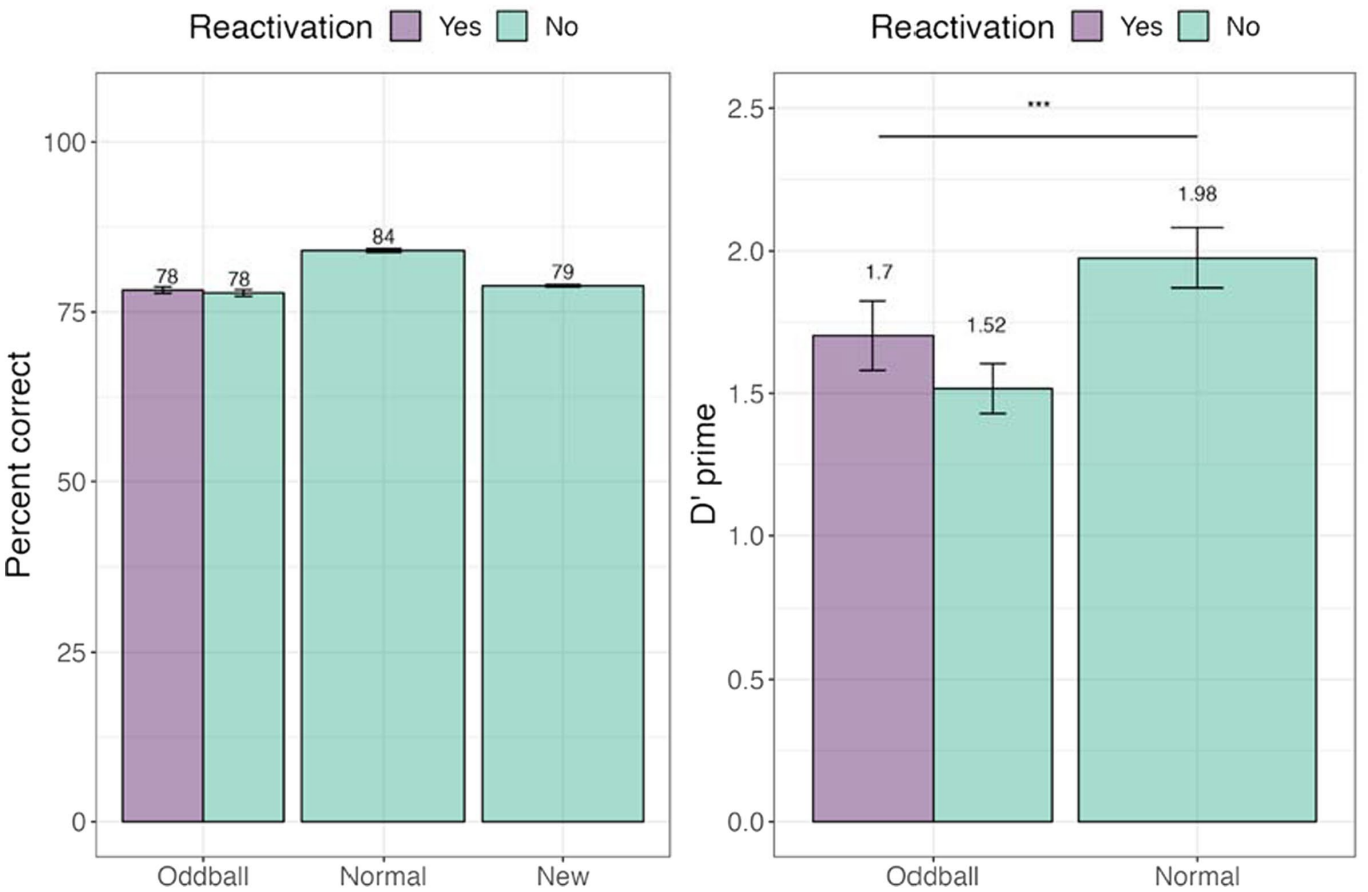
Percent and correct and d’-prime for the visual recognition task in Experiment 2. ***The significance of *p* < 0.001. Error bars represent standard errors of the mean.

We tested whether tagging led to an improvement in memorization of the images. Percent accuracy was calculated. We further calculated d’-prime (sensitivity) and c (criterion) measures (see Figure 8). A within-participant Analysis of Variance (ANOVA) was conducted with *stimuli* (reactivated oddball /oddball/normal) factor. We note that unlike the analysis used in Experiment 1, we did not have a separate reactivation factor, but instead compared the three stimuli types employed in Experiment 2’s design. There was a significant main effect of stimuli, *F*(1, 45) = 16.397, *p* = 0.0002. Pairwise contrasts among stimuli with holm adjustment for multiple comparisons revealed that participants were better at recognizing normal to oddball images, *t*(45) = −4.6, *p* = 0.0001. For full descriptive statistics see Supplementary Appendix III.

##### Recognition confidence

**Figure 9:**
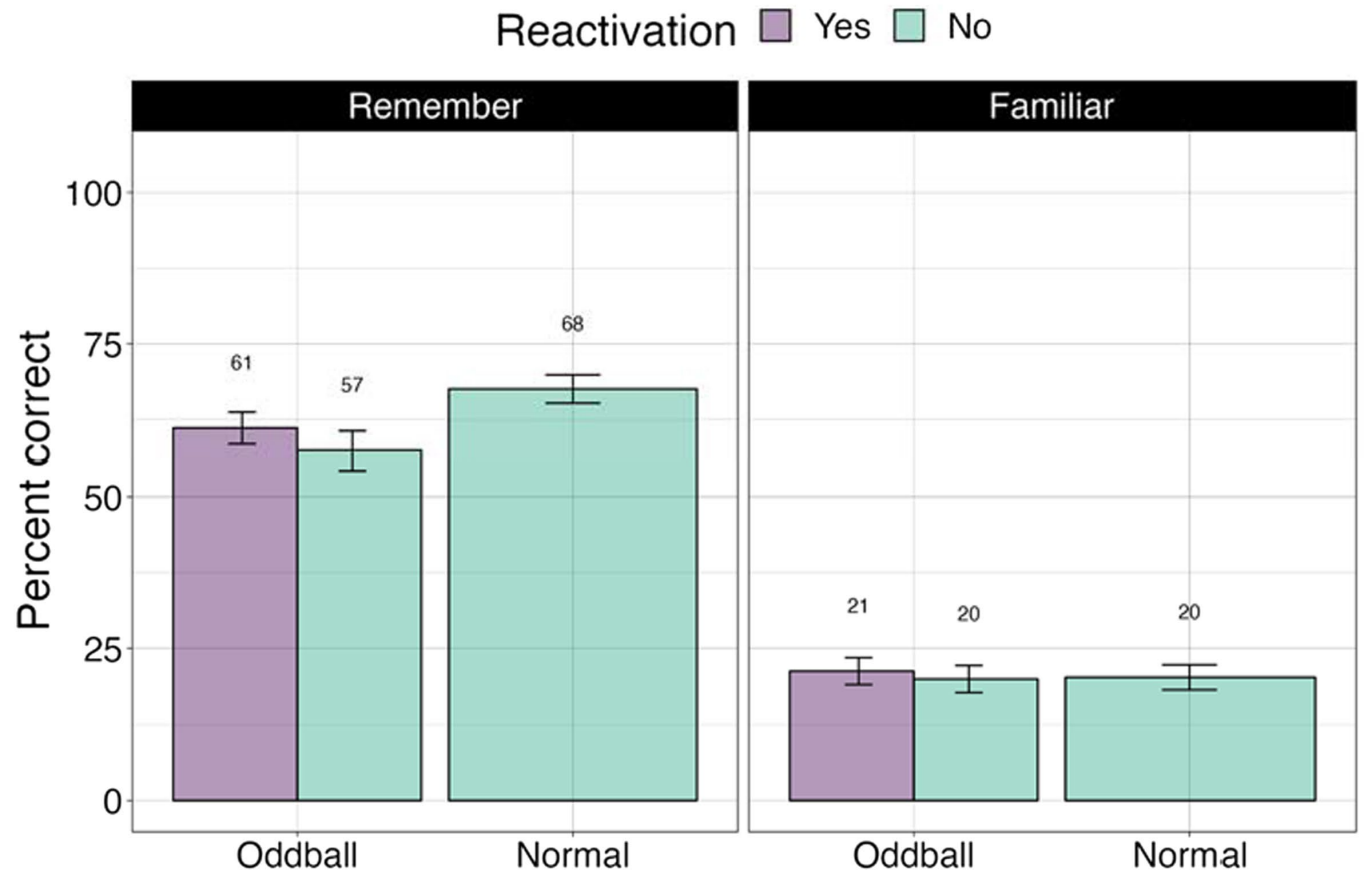
Percent correct for the visual recognition test by self-reported confidence levels in Experiment 2.

We conducted a 3×2 ANOVA, with *stimuli* (reactivated oddball/oddball/normal), and *confidence* (remember/familiar) factors. Fourteen participants were removed from this analysis due to missing values. There was a significant *stimuli* effect as found in the previous analysis, *F*(2, 62) = 11.44, *p* < 0.0001. Importantly, there was a significant *confidence* effect, *F*(1, 31) = 112.62, *p* < 0.0001. Participants were far more accurate for remembered compared familiar stimuli, mean difference = 36.5, *t*(49) = 10.6, *p* < 0.0001 (see Figure 9).

#### Location test

**Figure 10:**
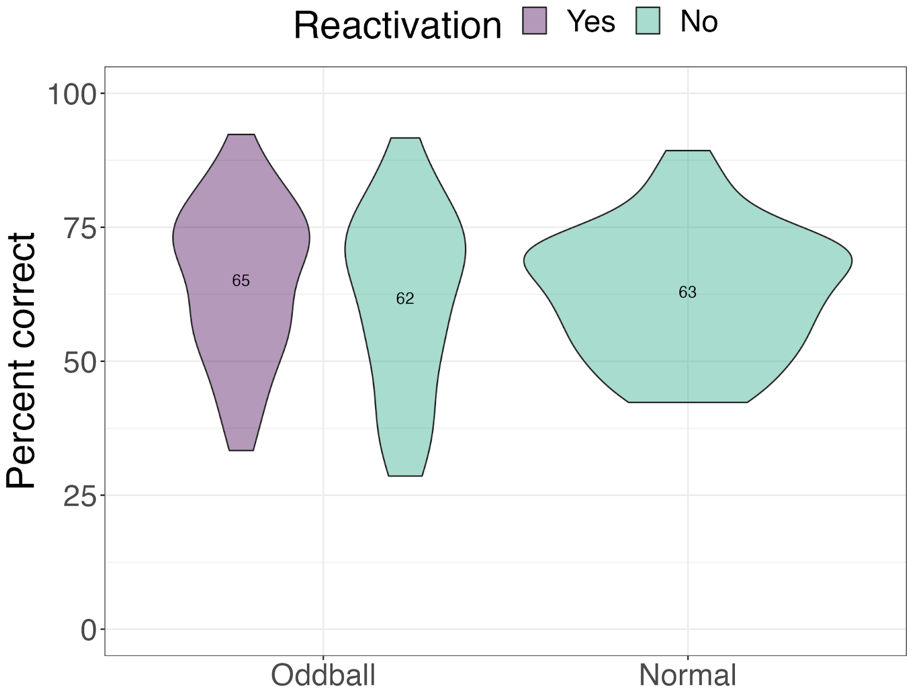
Mean accuracy for the visual recognition task in Experiment 2.

A one-way ANOVA revealed no significant differences across stimuli conditions (see Figure 10).

##### Location confidence

**Figure 11:**
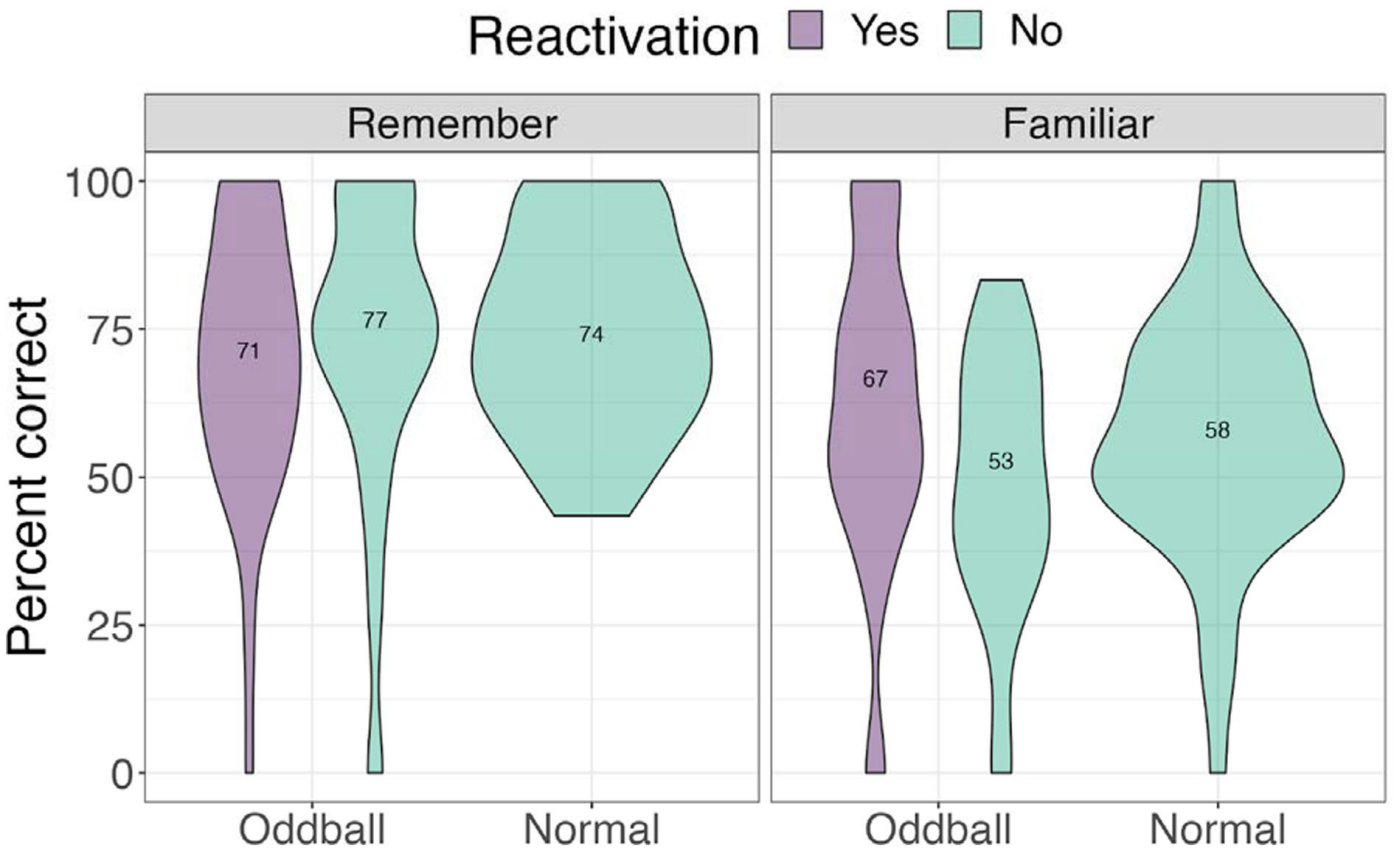
Mean accuracy for the visual recognition by different confidence levels task in Experiment 2.

We next examined accuracy and self-rated confidence level. we conducted a 3×2 ANOVA, with *stimuli* (reactivated oddball /oddball/normal) and *confidence* (remember/familiar) as factors. We note that 15 participants were removed from this analysis due to missing values (not all conditions had “remember” or “familiar” responses). There was a significant *confidence* effect, *F*(2, 60) = 26.948, *p* < 0.0001. Pairwise contrasts with holm correction reveal that participants were more accurate at recalling the location of “remember” of all stimuli types compared to “familiar” stimuli, mean difference = 12.5, *t*(49) = 3.46, *p* = 0.0045. Participants were also specifically better at recalling “remember” normal images compared to “familiar” normal images, mean difference = 19, *t*(30) = 5.19, *p* < 0.0001 (see Figure 11).

##### Cognitive ability

Similarly to Experiment 1, there are no meaningful correlations between general cognitive ability measured as accuracy on Raven’s advanced progressive matrices, sensitivity (d’ prime) in the recognition test, and accuracy in the location test.

### Summary

When participants were presented with visual images associated with a regular repeating sound or an oddball sound, they were better at remembering the normal images. Participants were also better at remembering normal images they rated as “remember” compared to those rated as “familiar,” suggesting a high association between memory accuracy and confidence ratings. This finding is different from results obtained in Experiment 1. We attribute this to a possible floor effect, whereby the memory task as a whole was difficult, suppressing possible benefits for sound tagging. One option is to make auditory encoding easier, for example, by presenting a complex sound that varies in several dimensions, such as loudness and pitch, from the normal sounds. A second option is to create an easier-to-learn association, such as pairing an image with a short mismatched melody. Another option is to utilize more potent sensory cues, such as odors that may trigger stronger attentional and emotional processing. These possibilities can be pursued in future studies.

## Discussion

We remember events and objects by their multisensory attributes—what things looked like, how they sound, where they were, how they made us feel. One way to strengthen memory is to replay parts of events (Oudiette et al., 2013). We propose that when retrieving information about these events shortly later, other sensory attributes are more easily accessed. In our study, participants were presented with a series of everyday objects and their sounds, with some objects having an unusual visual and auditory characteristic to render it more memorable. We found that reactivating one attribute (sound) of a particularly memorable event (an “oddball”) enhances memory for another attribute (location). Improved memory for spatial information occurred even though this information was encoded incidentally during initial presentation. A memory benefit occurred when oddball stimuli appeared randomly as opposed to being part of a sequence (e.g., a sequence of animals), presumably rendering the oddballs even more salient.

This study demonstrates the benefit of sensory reactivation during wakefulness and not sleep. Most studies exploring target memory reactivation focus on reactivation during sleep, when memory replay and consolidation processes often occur. Several studies suggest that reactivation can take place during wakeful periods as well (Oudiette et al., 2013; Tambini et al., 2017). Mednick et al. (2011) suggested that the brain seeks to opportunistically engage in consolidation processes during periods of reduced interference from external stimuli, whether during sleep or restful moments during the day. This is thought to be related to processing in the hippocampus, where when it is not involved in coding new events, consolidation of previous events occurs.

An interesting question is whether the same or different structures are involved in memory reactivation during wakefulness compared to sleep. A recent study on rat learning reveals that different structures support the creation of long-term object recognition representations (Sawangjit et al., 2022). During sleep, the hippocampus forms context-dependent representations, whereby different sensory attributes are binded together. During wakefulness, context-independent representations are formed by extra-hippocampal, striatal, and cortical regions. The hippocampus may also be forming parallel representations, if free from encoding new events. Wakeful reactivation may be particularly beneficial for attention-grabbing oddball events, where the object itself is of immediate interest. In contrast, reactivation during sleep boosts memory for event context, where context-representations are strengthened by hippocampal activation.

Another suggestion is that wakeful reactivation can work well for semantic tagging by capitalizing on existing long-term memories, whereas sensory tagging and reactivation (as in Experiment 2) requires sleep to strengthen new representations.

It is important to note that the time-course of TMR is not well addressed in our study. While, we assessed TMR in participants tested immediately after the wakeful reactivation, prior studies employed longer delays between reactivation and testing, for example, ~10 min (Oudiette et al., 2013), and 24 h (Tambini et al., 2017). The time interval between reactivation and test may be affecting different consolidation processes: At a shorter delay, short-term representations are modified, while at a longer delay, hippocampal representations are modified. Further research will be required to understand how changing the delays between encoding, reactivation and tests of recall impact results in our paradigm.

While previous studies presented information in the same modality during encoding, reactivation and test, we took a different approach, presenting *different sensory attributes of the same event* across encoding, reactivation and test. The memory benefits observed in this study support *dual coding* models proposing that multisensory stimuli are encoded by multiple systems, notably the visual and verbal systems (Clark and Paibio, 1991). Once a multisensory representation is formed, it can be accessed by its different unisensory components, either through direct links between sensory cortices, or *via* links to the same semantic representation (Driver and Noesselt, 2008; Shams and Seitz, 2008). We expand this framework by demonstrating that reactivating one sensory component during a consolidation period strengthens the entire multisensory representation, evident in superior memory for other sensory components.

*Audiovisual tagging* evokes existing long-term semantic representations. In this form of tagging, most objects appeared with their corresponding sounds (for example, animals with their vocals, and objects with the sound they omit). Oddballs were created by presenting some objects with mismatching sounds, with the aim of creating a deviation from existing associations. Reactivating mismatched sounds improved memory for oddball locations, compared to oddballs that were not reactivated. Wakeful reactivation is hence effective at modifying an oddball memory, a salient object with weaker context. In contrast, in a second experiment utilizing *sound tagging*, oddballs were created by capitalizing on a well-known perceptual mechanism, whereby irregular events draw attention. While most objects appeared with a single repeating sound, oddballs appeared with a unique novel sound. Here, no effect was found for reactivation on memory performance. One possibility is that wakeful reactivation is not effective in the complete absence of semantics, and reactivation during sleep may be needed to create new contextual representations. Another possibility is that overall low object recognition prevented a reactivation difference to manifest for object locations. In order to create stronger tagging, a future study can employ more complex sounds, a different sensory cue, or repeated stimuli presentations.

A limitation of Experiment 1 is an unequal number of participants between conditions. However, the conditions themselves each consisted of a within-participant design, allowing a direct comparison of the effect of reactivation on memory performance of the same sample of participants.

An exciting potential of TMR research is the potential to improve episodic memory in aging and amnesia with memory reactivation interventions (Fernández et al., 2022). While episodic memory tends to decline with age, MCI is characterized by a notable episodic memory impairment, without compromising everyday functioning. Fernández et al. (2022) found a benefit of a *reactivation intervention* on associative memory: Three groups of participants—adults, older adults, and MCI patients—learned new face-name pairs. After a day, half of the participants of each group were presented again with faces and the first letters of their names (to encourage active retrieval), while the other half did not. A day later, participants were tested on their memory for the face-name pairs. Across all groups, participants who underwent the reactivation intervention showed improved associative memory compared to the control condition. The memory benefit was particularly pronounced for MCI patients, who showed better memory for the face-name pairs, as well as memory for single faces or names. Reactivation is hence most potent for participants with the weakest memories. While reactivation is helpful for participants with varying degrees of episodic memory impairments originating in the function of the hippocampus, it may be that utilizing non-hippocampal representations can be even more effective. Future studies could target single-item memory, for example, reactivating oddball events such as an unusual name. Participants with a memory deficit or decline are hypothesized to show improved memory for such oddballs following wakeful reactivation, relaying on relatively intact non-hippocampal areas.

In conclusion, we tested the hypothesis that memories could be strengthened by coupling exposure events with sensory cues (either in a single or multiple modalities), and later reactivating these cues when participants are awake. One possible application of these results are ways to benefit memory for novel events. Such events could be new or unconventional educational material, new scientific findings, or new words in a language, such as technology-related words. These pieces of information could potentially be paired with different sensory cues, with these cues presented again during daily activities (such as listening to sounds when walking) to consolidate information. An exciting possibility is that TMR can be used in interventions to benefit those with memory concerns, such as older adults with memory declines. While future research in the field is certainly required, the beauty of multisensory memory interventions is that they are relatively simple to deploy and have shown some effectiveness to aid in memory encoding, consolidation, and recall.

## Data availability statement

The raw data supporting the conclusions of this article will be made available by the authors, without undue reservation.

## Ethics statement

The studies involving human participants were reviewed and approved by University of California Riverside, Human Subjects Review Board. The patients/participants provided their written informed consent to participate in this study.

## Author contributions

All authors designed the research. AG created and ran the experiments, analyzed the data, and wrote the manuscript with input from AS and LS. All authors contributed to the article and approved the submitted version.

## Conflict of interest

The authors declare that the research was conducted in the absence of any commercial or financial relationships that could be construed as a potential conflict of interest.

## Publisher’s note

All claims expressed in this article are solely those of the authors and do not necessarily represent those of their affiliated organizations, or those of the publisher, the editors and the reviewers. Any product that may be evaluated in this article, or claim that may be made by its manufacturer, is not guaranteed or endorsed by the publisher.
