# Supplementary material for: Improving memory for unusual events with wakeful reactivation
Source: Front Psychol. 2023 Mar 28;14:1092408. doi: 10.3389/fpsyg.2023.1092408 (PMC10086428; doi:10.3389/fpsyg.2023.1092408)
Supplement: Supplementary file 1 [file Data_Sheet_1.docx]

***Supplementary Material***

### Appendix I: Stimuli

*Visual stimuli by category:*

| **Category** | **Image** |
| --- | --- |
| Animals | Frog |
| Animals | Owl |
| Animals | Roster |
| Animals | Toy duck |
| Animals | Snake |
| Animals | Swan |
| Animals | Squirrel |
| Animals | Peacock |
| Music | Piano |
| Music | Bell |
| Music | Drum |
| Music | Small gong |
| Household items | Clock |
| Household items | Closet |
| Household items | Key |
| Household items | Door |
| Vehicles | Tank |
| Vehicles | Submarine |
| Vehicles | Car |
| Vehicles | Miniature ship |
| Kitchen | Coffee beans |
| Kitchen | Match box |
| Kitchen | Sink |
| Kitchen | Cup |
| Sport | Sleeping bag |
| Sport | Boxing gloves |
| Sport | Whistle |
| Sport | Machine game |

*Auditory oddballs examples:*

| Sounds |
| --- |
| Kissing |
| Laughing |
| Baby crying |
| Toy duck |
| Hands clapping |
| Keys |
| Heartbeat |
| Court hammer |
| Witch laugher |
| Snoring |
| Door closing |
| Bell ringing |
| Toilet flushing |
| Horse riding |
| Camera clicking |
| Sweeping |
| Water drop |
| Cleaning |
| Phone ringing |
| Jumping |
| Coins falling |

*Word search examples:*

###
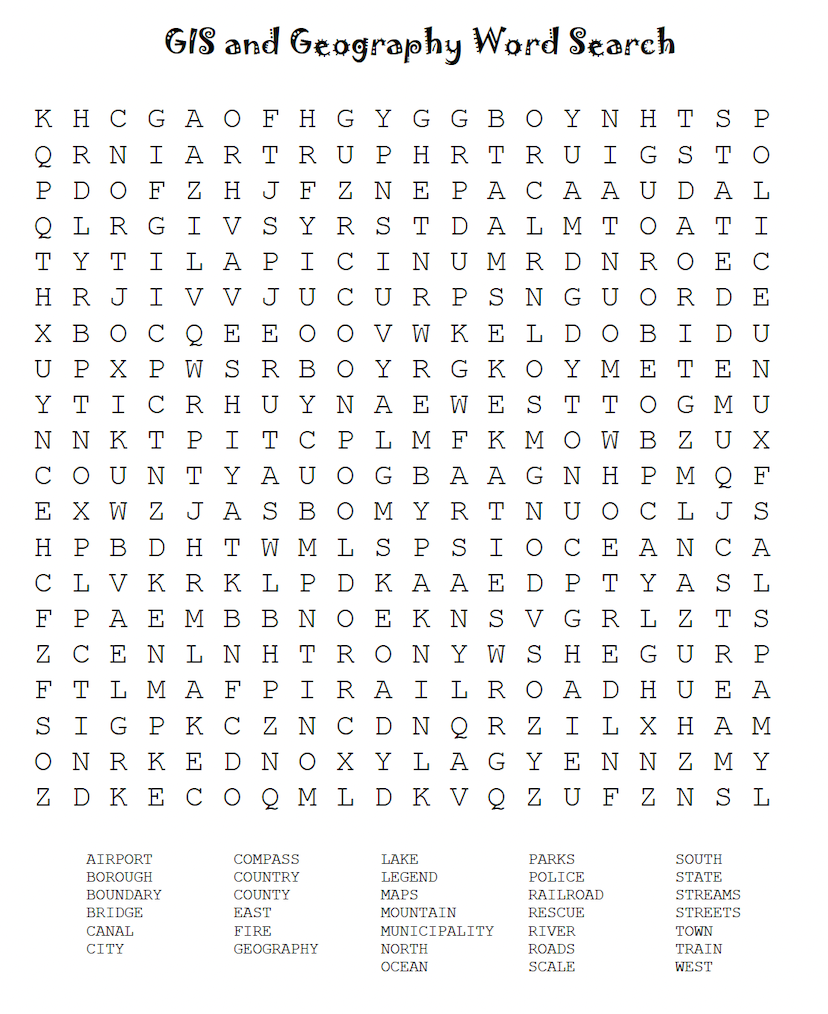


### Appendix II: Encoding

*Encoding tasks and outcomes:*

| **Experiment** | **Task** | **Trial Type** | **Outcome** | **Value** | **SD** |
| --- | --- | --- | --- | --- | --- |
| Audiovisual tagging | Encoding | Oddball | Percent correct | 45.2 | 23.4 |
| Audiovisual tagging | Encoding | Normal | Percent correct | 66.6 | 19.3 |
| Sound tagging | Encoding | Oddball | Percent correct | 79.6 | 18.0 |
| Sound tagging | Encoding | Normal | Percent correct | 79.7 | 13.6 |
| Audiovisual tagging | Encoding | Oddball | Reaction time (median) | 868.0 | 518.0 |
| Audiovisual tagging | Encoding | Normal | Reaction time (median) | 578.0 | 284.0 |
| Sound tagging | Encoding | Oddball | Reaction time (median) | 876.0 | 309.0 |
| Sound tagging | Encoding | Normal | Reaction time (median) | 583.0 | 184.0 |
| Audiovisual tagging | Surprise judgment | Oddball | Percent correct | 18.9 | 10.5 |
| Audiovisual tagging | Surprise judgment | Normal | Percent correct | 11.8 | 10.5 |
| Sound tagging | Surprise judgment | Oddball | Percent correct | 65.3 | 25.9 |
| Sound tagging | Surprise judgment | Normal | Percent correct | 20.2 | 25.9 |

### Appendix III: Signal detection

*Experiment 1: Audiovisual tagging*

| **Condition** | **d’-prime** | **c** |
| --- | --- | --- |
| Reactivated oddballs | 3.1 (0.146) | -1.02 (0.08) |
| Oddballs | 3.05 (0.191) | -1 (0.08) |
| Reactivated normal | 1.96 (0.119) | -0.45 (0.09) |
| Normal | 1.95 (0.133) | -0.44 (0.08) |

*Experiment 2: Sound tagging*

| **Condition** | **d’-prime** | **c** |
| --- | --- | --- |
| Reactivated oddballs | 1.7 (0.12) | -0.04 (0.05) |
| Oddballs | 1.5 (0.09) | 0.05 (0.06) |
| Normal | 1.97 (0.1) | -0.17 (0.05) |

### Appendix IV: General cognitive ability

*Experiment 1: Audiovisual tagging*

Correlations between accuracy on Raven’s advanced progressive matrices and memory measured obtained in the oddball memory task.

*Recognition test.* Correlations between accuracy on the Raven’s test and sensitivity (d’-prime).

*Location test.* Correlations between accuracy on the Raven’s test and accuracy.

| **Stimuli** | **Reactivation** | **Correlation with recognition performance** | **Correlation with location recall performance** |
| --- | --- | --- | --- |
| Oddballs | Yes | 0.07 | -0.03 |
| Oddballs | No | 0 | -0.04 |
| Normal | Yes | 0.067 | 0.08 |
| Normal | No | 0.1 | -0.076 |

### *Experiment 2: Sound tagging*

| **Stimuli** | **Correlation with recognition performance** | **Correlation with location recall performance** |
| --- | --- | --- |
| Reactivated oddballs | -0.1 | 0.23 |
| Oddballs | -0.08 | 0.03 |
| Normal | 0.06 | 0.1 |
